# Supplementary material for: Translating best practice into real practice: Methods, results and lessons from a project to translate an English sexual health survey into four Asian languages
Source: PLoS One. 2021 Dec 17;16(12):e0261074. doi: 10.1371/journal.pone.0261074 (PMC8682877; doi:10.1371/journal.pone.0261074)
Supplement: S1 Appendix — (DOCX) [file pone.0261074.s001.docx]

Dear [insert company/individual],

Curtin University

Tel: +61 8 9266 3668

Email: daniel.vujcich@curtin.edu.au

***BRIEF FOR TRANSLATORS – MiBSS***

Thank you for agreeing to translate the Migrant Blood-Borne Virus and Sexual Health Survey (MiBSS) into written **[insert language**]. In this letter, we aim to provide you with relevant contextual information about the study, details about our preferred approach to translation and specific instructions for completing the current task.

**BACKGROUND**

MiBSS is aimed at people born in Sub-Saharan Africa, South-East Asia and North-East Asia who have been living in Australia for at least 2 years. The aim of the survey is to understand how people think and act in relation to sexually transmissible infections and blood-borne viruses.

The survey will be completed by up to 1,800 adults across Australia. We are interested in recruiting people from a diverse range of genders, age groups, religions and ethnicities. The survey will be advertised at community venues including places of worship and cultural associations, at health services, through social media and at major events.

People will have an option to complete the survey online or with pen-and-paper. The survey will be available in the following written languages:

- English
- Traditional Chinese
- Simplified Chinese
- Modern Standard Arabic
- Khmer
- Vietnamese
- Karen.

**PROCESS FOR TRANSLATION**

We are using a team approach to translation which represents current best practice in survey research. Under this model, two independent **translators will complete the form in Attachment 1 (attached).** The two translated versions will then be compared by a review panel which will include bilingual participants who are familiar with survey research and/or the topic of sexually transmitted infections and blood-borne viruses.

Where the translations for particular items are inconsistent, the review panel will work together to decide on the most appropriate version to adopt in light of the specific needs of this study. The review panel may also contact the translators for further input in relation to inconsistencies.

As a final validation exercise, the agreed version of each translated survey will then be pre-tested on a small group of people who speak the language in question.

**GUIDELINES FOR TRANSLATING THIS SURVEY**

We ask that translators adhere the following principles when translating the English written version of the survey. These principles are reproduced/adapted from the *Guidelines for Best Practice in Cross-Cultural Surveys* which can be accessed at <https://ccsg.isr.umich.edu/index.php/chapters/translation-chapter>. See also <https://ccsg.isr.umich.edu/index.php/hidden-2/286-translation-overview-appendix-c-examples-of-common-causes-of-mistranslation>.

1. Translators should ask themselves ‘What does this survey item mean in the source questionnaire?’ and then put this understanding into the target language. They should produce translations that do not reduce or expand the information to the extent that the meaning or the concept of the original source question is no longer kept. However, ensuring a fully equivalent translation may sometimes turn out to be impossible, in particular if two languages do not have terms that match semantically or equivalent concepts at all. In these cases, the best possible approximation should be striven for and the lack of ‘full’ equivalence clearly noted.
2. It is recognised that, in contrast to English, many languages have complex pronoun systems that indicate number, gender, age, kinship or in-group/out-group relationships, and social status. It is essential to consider who a survey question is asking about in instances of otherwise ambiguous pronouns. Is it the respondent themselves, people in general, people like me, etc.? If the reference person differs between the source text and translation, this may lead to artifacts in the data that make comparison impossible.
3. Generally speaking, the English version of MiBSS expresses concepts in gender neutral ways. However, it is recognised that some languages have masculine and feminine versions of certain adjectives, nouns etc. In translating, the general aim should be to avoid excluding one of the genders while at the same time avoiding making a question too complicated or too difficult to ask by continuously repeating both genders. Any difficulties in striking this balance should be noted.
4. Translators should be mindful of clarity and fluency. In general, translators should do their best to produce questions that can readily be understood by the respondents. Writing questions that can be understood by the [target population](https://ccsg.isr.umich.edu/index.php/resources/advanced-glossary/target-population) requires not only taking into account usual target language characteristics but also involves taking into account the target group in terms of their age, education, etc. People of various origins should be able to understand the questionnaire in the intended sense without exerting particular effort.
5. Translators should use words that the average population can understand.
6. General consistency of tone is needed: this means that it is important to use the same style in the entire survey instrument, in terms of language register, politeness norms or level of difficulty.
7. Translators should try to be as concise and brief as possible in the translation and not put additional burden upon the respondent by making the translation unnecessarily long. Also, if forced by language constraints to spell out things more clearly in the target language than in the source language (e.g. two nouns rather than one noun; a paraphrase rather than an adverb), always keep the respondent burden to the minimum possible.
8. Translators should preserve the order of enumeration elements, listing multiple components in the target item in the same order as in the source item (unless otherwise specified in the Table include in Annexure 1).
9. Take care that the translations used do not convey any ambiguous / unintended connotations that would distort the results. In particular, we ask that you avoid translating in a way that reflects or contributes to social stigma and negative attitudes associated with concepts such as sex and sexuality, sexually transmitted infections and blood-borne viruses.
10. If it is not possible to translate in a way that preserves the meaning conveyed in the English version of the survey, translators should document reasons clearly in Annexure 1 (column labelled ‘Any comments’).
11. Translators should contact the Project Coordinator (details below) if they require further information about a particular survey item in order to produce an accurate translation.

**ATTACHMENTS**

1. TABLE FOR TRANSLATION
2. ENGLISH SURVEY (PAPER VERSION)

Yours sincerely,

Dr. Daniel Vujcich

Project Coordinator

[Daniel.Vujcich@curtin.edu.au](mailto:Daniel.Vujcich@curtin.edu.au)

0452 377 749

| **Footer - page numbers** | **1** | Translate if Arabic numerals are inappropriate |  |  | N/A |
| --- | --- | --- | --- | --- | --- |
|  | **2** |  |  |  |  |
|  | **3** |  |  |  |  |
|  | **4** |  |  |  |  |
|  | **5** |  |  |  |  |
|  | **6** |  |  |  |  |
|  | **7** |  |  |  |  |
|  | **8** |  |  |  |  |
|  | **9** |  |  |  |  |
|  | **10** |  |  |  |  |
|  | **11** |  |  |  |  |
|  | **12** |  |  |  |  |
|  | **Page** | N/A |  |  |  |
| **Footer – title** | **Migrant Blood-borne Virus and Sexual Health Survey** | N/A |  |  | N/A |
| **Question numbers** | **1.1** | Translate if Arabic numerals are inappropriate and/or list numbering format is inappropriate |  |  | N/A |
|  | **1.2** |  |  |  |  |
|  | **1.3** |  |  |  |  |
|  | **1.4** |  |  |  |  |
|  | **1.5** |  |  |  |  |
|  | **1.6** |  |  |  |  |
|  | **2.1** |  |  |  |  |
|  | **2.2** |  |  |  |  |
|  | **2.3** |  |  |  |  |
|  | **2.4** |  |  |  |  |
|  | **3.1** |  |  |  |  |
|  | **3.2** |  |  |  |  |
|  | **3.3** |  |  |  |  |
|  | **3.4** |  |  |  |  |
|  | **3.5** |  |  |  |  |
|  | **3.6** |  |  |  |  |
|  | **3.7** |  |  |  |  |
|  | **4.1** |  |  |  |  |
|  | **4.2** |  |  |  |  |
|  | **4.3** |  |  |  |  |
|  | **4.4** |  |  |  |  |
|  | **4.5** |  |  |  |  |
|  | **5.1** |  |  |  |  |
|  | **5.2** |  |  |  |  |
|  | **5.3** |  |  |  |  |
|  | **5.4** |  |  |  |  |
|  | **6.1** |  |  |  |  |
|  | **6.2** |  |  |  |  |
|  | **6.3** |  |  |  |  |
|  | **6.4** |  |  |  |  |
|  | **6.5** |  |  |  |  |
|  | **6.6** |  |  |  |  |
|  | **7.1** |  |  |  |  |
|  | **7.2** |  |  |  |  |
|  | **7.3** |  |  |  |  |
|  | **7.4** |  |  |  |  |
|  | **8.1** |  |  |  |  |
|  | **8.2** |  |  |  |  |
|  | **8.3** |  |  |  |  |
|  | **8.4** |  |  |  |  |
|  | **8.5** |  |  |  |  |
|  | **8.6** |  |  |  |  |
|  | **8.7** |  |  |  |  |
|  | **8.8** |  |  |  |  |
|  | **8.9** |  |  |  |  |
|  | **8.10** |  |  |  |  |
|  | **9.1** |  |  |  |  |
|  | **9.2** |  |  |  |  |
| **Frequently used terms** | Go to next question – Q | Q is used as an abbreviation for Question. If there is no equivalent, please just translate the full word “question” |  |  | N/A |
|  | Go to next page – Q |  |  |  |  |
|  | Go to next section – Q |  |  |  |  |
|  | Skip next question and go to Q | N/A |  |  |  |
|  | Yes | N/A |  |  |  |
|  | No | N/A |  |  |  |
|  | I don’t know | N/A |  |  |  |
|  | I can’t remember | N/A |  |  |  |
|  | Another reason (please specify): | N/A |  |  |  |
|  | I prefer not to answer | N/A |  |  |  |
| **Page 1: Explanatory text** | **This survey is ONLY for people living in Australia who:**   - **are aged 18 or over AND** - **were born in Sub-Saharan Africa, South-East Asia or North-East Asia (the BLUE REGIONS below)** | The text “BLUE REGIONS”, “ONLY” and “AND” have been put in capital letters for emphasis. If capital letters are not appropriate in the translation please use another technique for emphasising these words. |  |  | N/A |
| **Page 1: Map label (left hand side)** | ANY African country **other than** Western Sahara, Morocco, Tunisia, Algeria, Egypt or Libya | The text ANY has been put in capital letters and “other than” has been bolded for emphasis. If capital letters and b are not appropriate in the translation please use another technique for emphasising these words. |  |  | N/A |
| **Page 1: Map label (right hand side)** | ANY of the following places in Asia:  Brunei  Cambodia  China (mainland)  Hong Kong  Indonesia  Japan  Laos  Myanmar (Burma)  Timor-Leste  Macau  Malaysia  Mongolia  North Korea South Korea  Philippines  Singapore  Taiwan  Thailand  Vietnam | The text ANY has been put in capital letters for emphasis. If capital letters and underlining are not appropriate in the translation please use another technique for emphasising the word. |  |  | N/A |
| **Page 1: Blue shaded box** | The aim of this survey is to understand how overseas-born people living in Australia think and act in relation to sexually transmissible infections and blood-borne viruses. Please read the participant information sheet to find out more.   - I have read, or had read to me, the information statement and I understand its contents - I believe I understand the purpose, extent and possible risks of my involvement in this project. - I voluntarily consent to take part in this research project. - I have had an opportunity to ask questions by calling the **Project Coordinator, Dr Daniel Vujcich (08 9266 3668 or** [**daniel.vujcich@curtin.edu.au**](mailto:daniel.vujcich@curtin.edu.au)**)** and I am satisfied with the answers I have received. - I understand that this project has been approved by Curtin University Human Research Ethics Committee and will be carried out in line with the National Statement on Ethical Conduct in Human Research (2007). | N/A |  |  | N/A |
| **Page 1: Option** | I agree | N/A |  |  | N/A |
| **Page 1: Option** | I do not agree | N/A |  |  | N/A |
| **Page 1: Text in arrow** | Please tick one | Note if ‘ticks’ are not commonly used in the translated language please replace as appropriate throughout |  |  | N/A |
| **Page 2: Heading** | ABOUT THE SURVEY | N/A |  |  | N/A |
| **Page 2:**  **Explanatory text 1** | **This survey has nine sections and you may stop participating at any time:**   1. Questions about HIV / AIDS 2. Questions about other sexually transmitted infections 3. Questions about hepatitis B 4. Questions about hepatitis C 5. Questions about sexual activities and relationships 6. Questions about health care 7. Questions about travel 8. Questions about you 9. Questions about the survey | If there is no widely understood acronym for HIV/AIDS please feel free to translate the full terms here and below. |  |  | N/A |
| **Page 2:**  **Explanatory text 2** | **The survey will take around 10 minutes.** | N/A |  |  | N/A |
| **Page 2:**  **Explanatory text 3** | **Please do NOT refer to the internet or other resources to help you answer any questions – we are interested in what YOU know.** | The text NOT and YOU has been put in capital letters for emphasis. If capital letters and underlining are not appropriate in the translation please use another technique for emphasising the words. |  |  | N/A |
| **Page 2:**  **Explanatory text 4** | **This survey is anonymous. Your answers will be private.** | N/A |  |  | N/A |
| **Page 3:**  **Heading** | SECTION 1: QUESTIONS ABOUT HIV / AIDS | N/A |  |  | N/A |
| **Page 3: Explanatory text** | **This section asks you about your knowledge about human immunodeficiency virus (HIV) and / or acquired immunodeficiency syndrome (AIDS) and how HIV is passed on. This is not to test or quiz you; it is just to give us an understanding of what different community members know about HIV / AIDS.** | N/A |  |  | N/A |
| **Page 3: Q1.1** | Have you heard of HIV and / or AIDS? *(Tick one)* | N/A |  |  | N/A |
| **Page 3: Q1.2** | Is an HIV test done whenever someone has a blood test in Australia? *(Tick one)* | N/A |  |  | N/A |
| **Page 3:**  **Q1.3** | Is it safe to have sex without a condom with someone who has VERY LOW amounts of HIV in their blood? *(Tick one)* | N/A |  |  | N/A |
| **Page 3:**  **Q1.4** | Is there non-traditional medication available for people living with HIV so they can live a normal life? *(Tick one)* | The phrase “normal life” in this question is intended to indicate that the person can function like other people and do everyday things without being impeded by their HIV status.  The phrase “non-traditional medication” is intended to refer to pharmaceuticals, as opposed to herbal or alternative medicines. |  |  | N/A |
| **Page 3:**  **Q1.5** | Are there any medicines that people can take BEFORE SEX to protect themselves against HIV? *(Tick one)* | N/A |  |  | N/A |
| **Page 3:**  **Q1.6** | If you think there is a medicine that people can take before sex to protect themselves against HIV, what is the name of the medicine? *(Tick one)* | N/A |  |  | N/A |
| **Page 3: Q1.6 Box 1** | The name of the medicine is *(please specify):* | N/A |  |  | N/A |
| **Page 3: Q1.6 Box 2** | I don’t know the name of the medicine. | N/A |  |  | N/A |
| **Page 4: Heading** | **SECTION 2: QUESTIONS ABOUT OTHER SEXUALLY TRANSMITTED INFECTIONS** | N/A |  |  | N/A |
| **Page 4: Explanatory text** | **This section asks you about your knowledge of other sexually transmitted infections (STIs). This is not to test or quiz you; it is just to give us an understanding of what different community members know about STIs.** | Only use the equivalent of the acronym STI if it is likely to make sense in the translated language; otherwise, please just replace all references to the acronym with the translated version of the full term, sexually transmitted infections. |  |  | N/A |
| **Page 4:**  **Q2.1** | **Have you heard of the following sexually transmitted infections (STIs)?** *(Tick as many as you know)* | N/A |  |  | N/A |
| **Page 4:**  **Q2.1 Box 1** | Gonorrhoea | N/A |  |  | No need to translate the phonetic translation that appears in the English version.  No need to translate box 4 in the English survey - “I know some STIs but I don’t know what they are called in English” |
| **Page 4:**  **Q2.1 Box 2** | Syphilis | N/A |  |  |  |
| **Page 4:**  **Q2.1 Box 3** | Chlamydia | N/A |  |  |  |
| **Page 4:**  **Q2.1 Box 5** | I haven’t heard of any of them | N/A |  |  |  |
| **Page 4:**  **Q2.2** | Can a person have an STI without any symptoms? *(Tick one)* | N/A |  |  | N/A |
| **Page 4:**  **Q2.3** | Can a person with ONLY one sexual partner get an STI through sex? *(Tick one)* | The text ONLY has been put in capital letters for emphasis. If capital letters is not appropriate in the translation please use another technique for emphasising the word. |  |  |  |
| **Page 4:**  **Q2.4** | Can some STIs make it harder for women to get pregnant? *(Tick one)* | N/A |  |  | N/A |
| **Page 5:**  **Heading** | SECTION 3: QUESTIONS ABOUT HEPATITIS B | N/A |  |  | N/A |
| **Page 5:**  **Explanatory text** | **This section asks you about your knowledge of hepatitis B and how it is passed on.** | N/A |  |  | N/A |
| **Page 5: Q3.1** | Which of the following best describes you? *(Tick one)* | N/A |  |  | N/A |
| **Page 5: Q3.1 Box 1** | I have heard of hepatitis but I don’t know if it was hepatitis B or another type of hepatitis (for example, hepatitis A or hepatitis C) | N/A |  |  | N/A |
| **Page 5: Q3.1 Box 2** | I have heard of hepatitis B AND hepatitis C but I don’t know the difference between them | The text AND has been put in capital letters for emphasis. If capital letters is not appropriate in the translation please use another technique for emphasising the word. |  |  | N/A |
| **Page 5: Q3.1 Box 3** | I have **not** heard of hepatitis B | The text **not** has been put in bold for emphasis. If bold text is not appropriate in the translation please use another technique for emphasising the word. |  |  | N/A |
| **Page 5: Q3.1 Box 4** | I have heard of hepatitis B and I know what it is | N/A |  |  | N/A |
| **Page 5: Q3.2** | Is there a vaccine (injection) to stop people from getting hepatitis B? *(Tick one)* | N/A |  |  | N/A |
| **Page 5: Q3.3** | Can you get hepatitis B from swallowing food or water containing the faeces (poo) of an infected person? *(Tick one)* | There is no need to translate the informal ‘poo’ if people are likely to understand the translated term for ‘faeces’ |  |  | N/A |
| **Page 5: Q3.4** | Is there non-traditional medicine that can make the hepatitis B virus completely go away from a person's body?  (Tick one) | See above note re: meaning of “non-traditional medicine” |  |  | N/A |
| **Page 5: Q3.5** | Can hepatitis B normally be passed on through sex without a condom? *(Tick one)* | N/A |  |  | N/A |
| **Page 5: Q3.6** | Can hepatitis B normally be passed on by sharing a toothbrush or shaving razor? *(Tick one)* | N/A |  |  | N/A |
| **Page 5: Q3.7** | Can hepatitis B normally be passed on by sharing food with an infected person? *(Tick one)* | N/A |  |  | N/A |
| **Page 6: Heading** | **SECTION 4: QUESTIONS ABOUT HEPATITIS C** | N/A |  |  | N/A |
| **Page 6: Explanatory text** | **This section asks you about your knowledge of hepatitis C and how it is passed on.** | N/A |  |  | N/A |
| **Page 6: Q4.1** | Which of these statements best describes you? *(Tick one)* | N/A |  |  | N/A |
| **Page 6: Q4.1 Box 1** | I have heard of hepatitis C | N/A |  |  | N/A |
| **Page 6: Q4.1 Box 2** | I have **not** heard of hepatitis C | The text **not** has been put in bold for emphasis. If bold text is not appropriate in the translation please use another technique for emphasising the word. |  |  | N/A |
| **Page 6:**  **Q4.1 Instruction** | **Go to section 5 below – Q5.1** | N/A |  |  | N/A |
| **Page 6:**  **Q4.2** | Is there a vaccine (injection) to stop people from getting hepatitis C? *(Tick one)* | N/A |  |  | N/A |
| **Page 6:**  **Q4.3** | Is there non-traditional medicine that can make the hepatitis C virus completely go away from a person's body? *(Tick one)* | See above note re: meaning of “non-traditional medicine” |  |  | N/A |
| **Page 6:**  **Q4.4** | Can hepatitis C be passed on by sharing injecting equipment like needles and syringes? *(Tick one)* | N/A |  |  | N/A |
| **Page 6:**  **Q4.5** | Can someone get hepatitis C more than once in their lifetime? *(Tick one)* | N/A |  |  | N/A |
| **Page 6: section 5 heading** | **SECTION 5: QUESTIONS ABOUT SEXUAL ACTIVITIES AND RELATIONSHIPS** | N/A |  |  | N/A |
| **Page 6: Explanatory text** | This section asks you about personal information relating to sexual activities. We understand that this could be sensitive to some people. Your responses will be kept anonymous and confidential. | N/A |  |  | N/A |
| **Page 6:**  **Q5.1** | In the past twelve (12) months, how many people have you had sexual intercourse with (vaginal or anal)? *(Tick one)* | N/A |  |  | N/A |
| **Page 6:**  **Q5.1 Box 1** | I prefer not to answer | N/A |  |  | N/A |
| **Page 6:**  **Q5.1 Box 2** | 0 | N/A |  |  | N/A |
| **Page 6:**  **Q5.1 Box 3** | 1 | N/A |  |  | N/A |
| **Page 6:**  **Q5.1 Box 4** | 2 to 5 | N/A |  |  | N/A |
| **Page 6:**  **Q5.1 Box 5** | 6 to 10 | N/A |  |  | N/A |
| **Page 6:**  **Q5.1 Box 6** | 11 or more | N/A |  |  | N/A |
| **Page 6:**  **Q5.1 Box 7** | I can’t remember | N/A |  |  | N/A |
| **Page 7: Q5.2** | Which of the following best describes the MOST RECENT person you had sex with?*(Tick one)* | The text MOST RECENT has been put in capital letters for emphasis. If capital letters is not appropriate in the translation please use another technique for emphasising the word. |  |  | N/A |
| **Page 7: Q5.2 Box 1** | Someone you are in a committed relationship with (e.g. husband / wife, boyfriend / girlfriend) | N/A |  |  | N/A |
| **Page 7: Q5.2 Box 2** | A casual sex partner | By casual sex partner we mean a person who the survey respondent is NOT in a committed relationship with |  |  | N/A |
| **Page 7: Q5.2 Box 3** | A sex worker | Please chose a neutral, non-stigmatising translation for the term ‘sex worker’. |  |  | N/A |
| **Page 7: Q5.3** | Did you use a condom the MOST RECENT time you had sex? (Tick one) | The text MOST RECENT has been put in capital letters for emphasis. If capital letters is not appropriate in the translation please use another technique for emphasising the word. |  |  | N/A |
| **Page 7:**  **Q5.4** | Why did you NOT use a condom the MOST RECENT time you had sex? *(Tick as many as apply)* | The text MOST RECENT and NOT has been put in capital letters for emphasis. If capital letters is not appropriate in the translation please use another technique for emphasising the words. |  |  | N/A |
| **Page 7:**  **Q5.4 Box 1** | My partner and / or I did not have one | N/A |  |  | N/A |
| **Page 7:**  **Q5.4 Box 2** | My partner and / or I could not afford one | N/A |  |  | N/A |
| **Page 7:**  **Q5.4 Box 3** | My partner did not want to use one | N/A |  |  | N/A |
| **Page 7:**  **Q5.4 Box 4** | I did not want to use one | N/A |  |  | N/A |
| **Page 7:**  **Q5.4 Box 5** | My partner and / or I did not know where to get one | N/A |  |  | N/A |
| **Page 7:**  **Q5.4 Box 6** | My partner doesn’t like the way they feel | N/A |  |  | N/A |
| **Page 7:**  **Q5.4 Box 7** | I don’t like the way they feel | N/A |  |  | N/A |
| **Page 7:**  **Q5.4 Box 8** | My partner or I was trying to get pregnant | N/A |  |  | N/A |
| **Page 7:**  **Q5.4 Box 9** | It is against my or my partner’s culture or religion | N/A |  |  | N/A |
| **Page 7:**  **Q5.4 Box 10** | My partner and I don’t have any illnesses that can be passed on through sex | N/A |  |  | N/A |
| **Page 7:**  **Q5.4 Box 11** | My partner and I trust each other | N/A |  |  | N/A |
| **Page 7: heading** | SECTION 6: QUESTIONS ABOUT HEALTH CARE | N/A |  |  | N/A |
| **Page 7: explanatory text** | **This section is about your experience of and access to health care for sexually transmitted infections (STIs) and blood-borne viruses (BBVs). BBVs include hepatitis B and C.** | Only use the equivalent of the acronym BBV if it is likely to make sense in the translated language; otherwise, please just replace all references to the acronym with the translated version of the full term, blood-borne viruses |  |  | N/A |
| **Page 7:**  **Q 6.1** | When did you have your most recent test for HIV, hepatitis B, hepatitis C or any sexually transmitted infections (we call these STI and / or BBV tests)? *(Tick one)* | N/A |  |  | N/A |
| **Page 7:**  **Q 6.1 Box 1** | Less than 12 months ago | N/A |  |  | N/A |
| **Page 7:**  **Q 6.1 Box 2** | 1 to 2 years ago | N/A |  |  | N/A |
| **Page 7:**  **Q 6.1 Box 3** | More than 2 years ago | N/A |  |  | N/A |
| **Page 7:**  **Q 6.1 Box 4** | I have never been tested | N/A |  |  | N/A |
| **Page 8: Q6.2** | Why did you NOT have an STI and / or BBV test in the last two years? *(Tick as many as apply)* | The text NOT has been put in capital letters for emphasis. If capital letters is not appropriate in the translation please use another technique for emphasising the word. |  |  | N/A |
| **Page 8: Q6.2 Box 1** | I did not do anything to put me at risk | N/A |  |  | N/A |
| **Page 8: Q6.2 Box 2** | I was too embarrassed | N/A |  |  | N/A |
| **Page 8: Q6.2 Box 3** | I could not afford extra tests | N/A |  |  | N/A |
| **Page 8: Q6.2 Box 4** | I didn’t know where to get one | N/A |  |  | N/A |
| **Page 8: Q6.2 Box 5** | I was scared about the result | N/A |  |  | N/A |
| **Page 8: Q6.2 Box 6** | I don't like needles / blood tests | The term ‘needles’ here refers to needles used in medical procedures. |  |  | N/A |
| **Page 8: Q6.2 Box 7** | I did not have any symptoms | N/A |  |  | N/A |
| **Page 8: Q6.2 Box 8** | I did not have the time to get tested | N/A |  |  | N/A |
| **Page 8: Q6.2 Box 9** | I did not think it was important | N/A |  |  | N/A |
| **Page 8: Q6.2 Box 10** | I couldn’t get to a service / clinic | N/A |  |  | N/A |
| **Page 8:**  **Q6.2 instruction** | If you answered this question (Q6.2) please go straight to Q6.5 | N/A |  |  | N/A |
| **Page 8:**  **Q6.3** | What was your MOST RECENT STI and / or BBV test for? *(Tick as many as apply)* | The text MOST RECENT has been put in capital letters for emphasis. If capital letters is not appropriate in the translation please use another technique for emphasising the word. |  |  | N/A |
| **Page 8:**  **Q6.3 BOX 1** | HIV | N/A |  |  | N/A |
| **Page 8:**  **Q6.3 BOX 2** | Chlamydia and / or gonorrhoea | N/A |  |  | N/A |
| **Page 8:**  **Q6.3 BOX 3** | Syphilis | N/A |  |  | N/A |
| **Page 8:**  **Q6.3 BOX 4** | Hepatitis B and / or hepatitis C | N/A |  |  | N/A |
| **Page 8:**  **Q6.3 BOX 5** | I don't know – it was a blood test | N/A |  |  | N/A |
| **Page 8:**  **Q6.3 BOX 6** | I don't know – it was a urine test | N/A |  |  | N/A |
| **Page 8:**  **Q6.3 BOX 7** | I don't know – it was a blood and urine test | N/A |  |  | N/A |
| **Page 8:**  **Q6.3 BOX 8** | Other (please specify): | N/A |  |  | N/A |
| **Page 8: Q6.4** | What was the reason for your MOST RECENT STI and / or BBV test? *(Tick as many as apply)* | The text MOST RECENT has been put in capital letters for emphasis. If capital letters is not appropriate in the translation please use another technique for emphasising the word. |  |  | N/A |
| **Page 8: Q6.4 Box 1** | I was applying for permanent residency | N/A |  |  | N/A |
| **Page 8: Q6.4 Box 2** | I had a new sexual partner | N/A |  |  | N/A |
| **Page 8: Q6.4 Box 3** | I shared injecting equipment with someone | Here we are referring to drug injecting equipment |  |  | N/A |
| **Page 8: Q6.4 Box 4** | Something happened that may have put me at risk | N/A |  |  | N/A |
| **Page 8: Q6.4 Box 5** | I was pregnant and had a check up | N/A |  |  | N/A |
| **Page 8: Q6.4 Box 6** | I was getting contraception / birth control | N/A |  |  | N/A |
| **Page 8: Q6.4 Box 7** | My doctor / nurse suggested it | N/A |  |  | N/A |
| **Page 8: Q6.4 Box 8** | My doctor / nurse just did it | N/A |  |  | N/A |
| **Page 8: Q6.4 Box 9** | I wanted to know if I had a sexually transmitted infection or a blood-borne virus | N/A |  |  | N/A |
| **Page 8: Q6.4 Box 10** | I had symptoms | N/A |  |  | N/A |
| **Page 8: Q6.4 Box 11** | It was part of my regular health check | N/A |  |  | N/A |
| **Page 8: Q6.4 Box 12** | I like to get regular STI / BBV tests | N/A |  |  | N/A |
| **Page 8: Q6.4 Box 13** | It was a requirement for my work / study | N/A |  |  | N/A |
| **Page 8: Q6.5** | **How would you feel if a doctor in Australia offered you STI and BBV tests during an appointment without you requesting any of these tests?** *(Tick any that apply)* | N/A |  |  | N/A |
| **Page 8: Q6.5 Box 1** | Offended – why are they asking me? | N/A |  |  | N/A |
| **Page 8: Q6.5 Box 2** | Worried – do they think I have an illness? | N/A |  |  | N/A |
| **Page 8: Q6.5 Box 3** | Surprised – I wasn’t expecting that | N/A |  |  | N/A |
| **Page 8: Q6.5 Box 4** | Okay – STI and BBV testing is normal | N/A |  |  | N/A |
| **Page 8: Q6.5 Box 5** | Relieved – now I don't have to ask for the tests | N/A |  |  | N/A |
| **Page 8: Q6.5 Box 6** | Embarrassed – I'd rather not talk about these things | N/A |  |  | N/A |
| **Page 8: Q6.5 Box 7** | Other (please specify): | N/A |  |  | N/A |
| **Page 9: Q6.6** | If a close friend in Australia told you that they were going to get tested for STIs and BBVs, how would you feel? *(Tick any that apply)* | Here we are referring to a platonic (non-sexual) friendship |  |  | N/A |
| **Page 9:**  **Q6.6 Box 1** | Fine – it’s none of my business | N/A |  |  | N/A |
| **Page 9:**  **Q6.6 Box 2** | Shocked – I didn’t think they would need to get tested | N/A |  |  | N/A |
| **Page 9:**  **Q6.6 Box 3** | Proud – it’s a responsible thing to do | N/A |  |  | N/A |
| **Page 9:**  **Q6.6 Box 4** | Supportive – I am here if they need my help | N/A |  |  | N/A |
| **Page 9:**  **Q6.6 Box 5** | Worried – I hope they are okay | N/A |  |  | N/A |
| **Page 9:**  **Q6.6 Box 6** | Disappointed – they must have done something wrong | N/A |  |  | N/A |
| **Page 9:**  **Q6.6 Box 7** | Other (please specify): | N/A |  |  | N/A |
| **Page 9:**  **Heading** | SECTION 7: QUESTIONS ABOUT TRAVEL | N/A |  |  | N/A |
| **Page 9: Explanatory text** | **This section is about your travel to other countries after migrating to Australia. This can be ANY country (not just your country of birth). Your responses will only be used for the purpose of this research.** | N/A |  |  | N/A |
| **Page 9:**  **Q7.1** | Since January 2018, how many times have you visited any country outside of Australia? *(Tick one)* | N/A |  |  | N/A |
| **Page 9:**  **Q7.1 Box 1** | 0 | N/A |  |  | N/A |
| **Page 9:**  **Q7.1 Box 2** | 1 to 2 times | N/A |  |  | N/A |
| **Page 9:**  **Q7.1 Box 3** | 3 to 4 times | N/A |  |  | N/A |
| **Page 9:**  **Q7.1 Box 4** | 5 to 6 times | N/A |  |  | N/A |
| **Page 9:**  **Q7.1 Box 5** | 7 or more times | N/A |  |  | N/A |
| **Page 9:**  **Q7.1 Box 6** | I can’t remember the number of times | N/A |  |  | N/A |
| **Page 9:**  **Q7.2** | On any of the overseas visits since January 2018, did you have sexual intercourse with at least one person who lives outside of Australia? *(Tick one)* | N/A |  |  | N/A |
| **Page 9:**  **Q7.3** | How often did you use condoms with any overseas sexual partner(s) since January 2018? *(Tick one)* | N/A |  |  | N/A |
| **Page 9:**  **Q7.3 Box 1** | Always | N/A |  |  | N/A |
| **Page 9:**  **Q7.3 Box 2** | Sometimes | N/A |  |  | N/A |
| **Page 9:**  **Q7.3 Box 3** | Never | N/A |  |  | N/A |
| **Page 9:**  **Q7.4** | Since January 2018, have you had sex with a sex worker while overseas? *(Tick one)* | Please chose a neutral, non-stigmatising translation for the term ‘sex worker’. |  |  | N/A |
| **Page 10: Heading** | SECTION 8: QUESTIONS ABOUT YOU | N/A |  |  | N/A |
| **Page 10: Explanatory text** | **Questions in this section relate to personal information about you. Your responses will only be used for the purpose of this research.** | N/A |  |  | N/A |
| **Page 10: Q8.1** | How old are you? *(Tick one)* | N/A |  |  | N/A |
| **Page 10: Q8.1 Box 1** | 18 to 29 years | N/A |  |  | N/A |
| **Page 10: Q8.1 Box 2** | 30 to 39 years | N/A |  |  | N/A |
| **Page 10: Q8.1 Box 3** | 40 to 49 years | N/A |  |  | N/A |
| **Page 10: Q8.1 Box 4** | 50 to 59 years | N/A |  |  | N/A |
| **Page 10: Q8.1 Box 5** | 60 years or over | N/A |  |  | N/A |
| **Page 10: Q8.1 Box 6** | Prefer not to answer | N/A |  |  | N/A |
| **Page 10: Q8.2** | How do you currently describe your gender identity?  (e.g. woman, man, transgender) | We prefer ‘gender’ as distinct from ‘biological sex’; however it is recognized that there may not be an equivalent concept in all languages. Please indicate if this is the case.  For ‘transgender’ please use a neutral, non-stigmatising translation. If none is available please indicate this in the notes. |  |  | N/A |
| **Page 10: Q8.2** | Please specify: | N/A |  |  | N/A |
| **Page 10: Q8.3** | What is the postcode in which you live? | N/A |  |  | N/A |
| **Page 10: Q8.4** | What your residency status in Australia? *(Tick one)* | N/A |  |  | N/A |
| **Page 10: Q8.4 Box 1** | I am a permanent resident / citizen | N/A |  |  | N/A |
| **Page 10: Q8.4 Box 2** | Temporary – student visa | N/A |  |  | N/A |
| **Page 10: Q8.4 Box 3** | Temporary – work visa | N/A |  |  | N/A |
| **Page 10: Q8.4 Box 4** | Temporary – holiday / tourist visa | N/A |  |  | N/A |
| **Page 10: Q8.4 Box 5** | Temporary – partner visa | N/A |  |  | N/A |
| **Page 10: Q8.4 Box 6** | Other (please specify): | N/A |  |  | N/A |
| **Page 10: Q8.5** | In which country were you born? | N/A |  |  | N/A |
| **Page 10: Q8.6** | How many years have you been living in Australia in total? | N/A |  |  | N/A |
| **Page 10: Q8.7** | What is your religion? | N/A |  |  | N/A |
| **Page 10: Q8.7 Box 1** | Christian | N/A |  |  | N/A |
| **Q8.7 Box 2** | Buddhist | N/A |  |  | N/A |
| **Q8.7 Box 3** | No religion | N/A |  |  | N/A |
| **Q8.7 Box 4** | Other (please specify): | N/A |  |  |  |
| **Q8.7 Box 5** | Jewish | N/A |  |  | N/A |
| **Q8.7 Box 6** | Muslim | N/A |  |  | N/A |
| **Page 10:**  **Q8.8** | Which of the following types of people are you sexually attracted to at the moment? *(Tick as many as apply)* | N/A |  |  | N/A |
| **Page 10: Q8.8 Box 1** | Men | N/A |  |  | N/A |
| **Page 10: Q8.8 Box 2** | Women | N/A |  |  | N/A |
| **Page 10: Q8.8 Box 3** | Transgender people | For ‘transgender’ please use a neutral, non-stigmatising translation. If none is available please indicate this in the notes. |  |  | N/A |
| **Page 10: Q8.8 Box 4** | Not sure | N/A |  |  | N/A |
| **Page 10: Q8.9** | What are the main languages you speak at home? | N/A |  |  | N/A |
| **Page 11: Q8.10** | Which cultural / ethnic group categories do you identify with? | N/A |  |  | N/A |
| **Page 11 note** | For instance, you might identify with:   - One group of people within your country of birth (e.g. Zulu, Hmong) - Your ancestral heritage (e.g. Indian-Malaysian or Chinese-Vietnamese) - People from another place you have lived (e.g. British) | If Zulu and Hmong are not likely to be meaningful in the translated language please delete |  |  |  |
| **Page 11:**  **Header** | SECTION 9: QUESTIONS ABOUT THIS SURVEY | N/A |  |  | N/A |
| **Page 11:**  **Explanatory text** | This section is about your experience completing this survey.  It will help us understand whether we need to make any changes to this survey in the future. | N/A |  |  | N/A |
| **Page 11: Q9.1** | Please read each statement below. Check the response that best indicates your level of agreement with each statement. The statements relate to your feelings about doing this survey. *(Tick one for each statement)* | N/A |  |  | N/A |
| **Page 11: Q9.1 – Option 1** | Strongly Agree | N/A |  |  | N/A |
| **Page 11: Q9.1 – Option 2** | Agree | N/A |  |  | N/A |
| **Page 11: Q9.1 – Option 3** | Disagree | N/A |  |  | N/A |
| **Page 11: Q9.1 – Option 4** | Strongly disagree | N/A |  |  | N/A |
| **Page 11: Q9.1 – Statement 1** | I felt upset* | The asterisk is to indicate that there is a note to this statement. If an asterisk is not appropriate please indicate a suitable alternative |  |  |  |
| **Page 11: Q9.1 – Statement 2** | I felt embarrassed | N/A |  |  | N/A |
| **Page 11: Q9.1 – Statement 3** | The survey was too long | N/A |  |  | N/A |
| **Page 11: Q9.1 – Statement 4** | I found it hard to understand some questions / words | N/A |  |  | N/A |
| **Page 11: Q9.1 – Statement 5** | I felt comfortable completing the survey | N/A |  |  | N/A |
| **Page 11: Q9.1 – note** | *If this survey has left you feeling upset please visit our webpage for contact details of services that can support you: mibss.org | The asterisk is to indicate that this is a note. If an asterisk is not appropriate please indicate a suitable alternative. |  |  | N/A |
| **Page 11- Q9.2** | Do you have any other comments or feedback about this survey? (Please specify) | N/A |  |  | N/A |
| **Page 11 – concluding remarks** | We are grateful for your time.  If you would like to know the progress of the MiBSS study you can check our website for regular updates: mibss.org   If you would like more information about HIV, STIs or BBVs we recommend you visit the website [allgood.org.au](http://allgood.org.au/).  All Good focuses on key information and provides tools and referral pathways to testing and treatment. Information is provided in 17 different languages, including English, in both text and spoken word recordings. | N/A |  |  | N/A |
|  | END OF SURVEY |  |  |  |  |
| **GENERAL** | Please provide general comments to assist us with appropriate formatting. For instance:   - Does the translated text need to be presented left to right or right to left? - Is the configuration of the checkboxes appropriate? - Is the use of columns appropriate? | These are questions for the translator. |  |  |  |
