# Supplementary material for: Translating best practice into real practice: Methods, results and lessons from a project to translate an English sexual health survey into four Asian languages
Source: PLoS One. 2021 Dec 17;16(12):e0261074. doi: 10.1371/journal.pone.0261074 (PMC8682877; doi:10.1371/journal.pone.0261074)
Supplement: Attachment — Submitted filename: Response to reviewers.docx [file pone.0261074.s004.docx]

July 2021

Dear Editors,

**Translating best practice into real practice: methods, results and lessons from a project to translate an English sexual health survey into four Asian languages**

Thank you for your considered and helpful feedback on our manuscript. We have reproduced and responded to each item of feedback below.

1. **ASSESSING EQUIVALENCE**

Feedback:

- *The team approach TRAPD for translation is systematic, but no statistical method conducted to assess the equivalence such as content validity test (CVI). Is it possible to add or combine statistical method to increase the validity of the translation?*

Response:

While statistical techniques such as exploratory and confirmatory factor analysis are commonly used to test equivalence of clinical assessment instruments and screening tools, they are less common in the context of population surveys geared towards measuring knowledge, attitudes and behaviours. The [Cross-Cultural Survey Guidelines](https://ccsg.isr.umich.edu/chapters/pretesting/) state that: “When multiple languages are used in the same survey, *pretesting* the different language versions is an essential part of ensuring [measurement equivalence](https://ccsg.isr.umich.edu/glossary/measurement-equivalence/) and cultural and cross-cultural equivalence” (emphasis added). Pretesting data are typically collected using qualitative methods such as cognitive interviews, focus group discussions and behaviour coding.

Assessing equivalence through the use of qualitative methods is the approach taken in a number of population surveys, including:

- The National Health Interview Survey (Cancer Control Module) and the California Health Interview Survey (Willis G, Stapleton M.S., Leven K., Norberg A., Stark D., Forsyth B., Brick P.D., Berrigan D., Thompson F.E., Lawrence D., Hartman A. Evaluation of a multistep survey translation process. In Harkness J., et al (eds). Survey methods in multinational, multiregional, and multicultural contexts. London, John Wiley & Sons. 2010);
- The Tobacco Use Survey component of the Current Population Survey (Forsyth B., Kudela M.S., Levin K., Lawrence D., Willis G. Methods for translating an English-language survey questionnaire on tobacco use in Mandarin, Cantonese, Korean, and Vietnamese. *Field Methods*. 2007;19(3):264-283);
- US Household Food Security Survey (Kwan C.M., Napoles A.M., Chou J., Seligman H.K. Development of a conceptually equivalent Chinese-language translation of the US Household Food Security Survey Module for Chinese immigrants to the USA. *Public Health Nutr.* 2015;18(2):242-250);
- An 11-language survey conducted by the European School on Interdisciplinary Tinnitus Research (Biswas R., Lugo A., Gallus S., Akeroyd M.A., Hall D.A. Standardized questions in English for estimating tinnitus prevalence and severity, hearing difficulty and usage of healthcare resources, and their translation into 11 European languages. *Hear. Res.* 2019;377:330-338); and
- The Parent Attitudes about Childhood Vaccines Survey (Cunningham R.M., Kerr G.B., Orobio J., et al Development of a Spanish version of the parent attitudes about childhood vaccines survey. *Hum. Vaccines Immunother.* 2019;15(5):1106-1110).

In our Discussion we note that:

… [A]dditional and innovative techniques for quality appraisal are prudent to check the quality of translations. For instance, in the context of our study, we plan to compare results obtained from English-language and target-language surveys, controlling for factors such as country of birth, length of time in Australia and gender. Any differences in responses may serve as an indication that the translated version did not convey the same meaning as the English version of the survey, prompting further investigation into the adequacy of the translation.

However, we consider that actually undertaking this analysis lies beyond the scope of this manuscript, the explicit aim of which is to document the process and results of applying the conventional TRAPD method.

2. **SAMPLE SIZE**

Feedback:

- *In the pretesting phase, the sample size of Vietnamese was 20, but other migrant communities were 3 or 4. Why was there a great variation in the sample size in different groups?*
- *In your Methods section, please provide a justification for the sample size used in your study during the pre-testing of the translated questionnaire, including any relevant power calculations (if applicable).*

Response:

Decisions around sample size for pretesting must be read in context. In the Introduction, we state: “The project formed one part of study to ascertain the feasibility of conducting a periodic national survey in Australia to measure migrants’ knowledge, attitudes and practices in relation to sexual health and blood-borne viruses.” We have further clarified the study’s emphasis on investigating feasibility in the first paragraph of the Methods section:

“Due to budgetary constraints, the English-language survey could only be translated into a limited number of languages. As this was a feasibility study, four languages were chosen to gain insight into the complexities of multi-language survey development and administration” (underlined text signifies revision).

Additionally, we have redrafted the pretesting sub-section of the Methods to make it clear that the sampling strategy for pretesting was pragmatic and primarily guided by the availability of resources:

The minimum target sample size for the pilot survey (in any language) was 1,116 respondents equally divided between the three regions of birth (Sub-Saharan Africa, North East Asia and South-East Asia) to detect regional differences at a significance level of 5% and 90% power. Based on available Census data on country of birth and English-language proficiency, it was estimated that:

- 82.00% (n=305) of North East Asian-born respondents would be from a country in which Chinese was the dominant language and that, assuming all spoke some Chinese, 25.9% (n=79) would be likely to not speak English well or at all;
- 25.21% (n=94) of South East Asian-born respondents would be from Vietnam and that, assuming all spoke Vietnamese, 31.50% (n=30) would be likely to not speak English well or at all;
- 3.81% (n=14) of South East Asian-born respondents would be from Cambodia and that, assuming all spoke Khmer, 33.50% (n=5) would be likely to not speak English well or at all;
- 3.75% (n=14) of South-East Asian-born respondents would be from Myanmar and less than 10% would speak Karen (proportion of those who would be likely to speak English not well or not at all is unknown) [20,21].

Given these small numbers, the limited availability of resources for pretesting, the exploratory nature of the feasibility study, and the fact that multiple quality checking methods were built into the study design (e.g. two independent translations, review and adjudication), a pragmatic decision was made to only pretest translations on small samples. Pretest participants were recruited using convenience sampling by two members of the research team who had experience working with these migrant communities. The size of the samples varied by community – Chinese (n=3), Vietnamese (n=20), Khmer (n=4) and Karen (n=3). The larger sample size for Vietnamese pretesting was opportunistic. While all pretest participants were fluent in the survey language being pretested, no other demographic characteristics were recorded.

In the Discussion section we have also now expressly noted our approach to pretesting as a limitation (see line 400 of revised transcript).

3. **ETHICS**

Feedback:

- *Please provide additional details regarding participant consent. In the ethics statement in the Methods and online submission information, please ensure that you have specified whether consent was written or verbal/oral. If consent was verbal/oral, please specify: 1) whether the ethics committee approved the verbal/oral consent procedure, 2) why written consent could not be obtained, and 3) how verbal/oral consent was recorded. If your study included minors, please state whether you obtained consent from parents or guardians in these cases. If the need for consent was waived by the ethics committee, please include this information.*
- *Please include your full ethics statement in the ‘Methods’ section of your manuscript file. In your statement, please include the full name of the IRB or ethics committee who approved or waived your study, as well as whether or not you obtained informed written or verbal consent. If consent was waived for your study, please include this information in your statement as well.*

Response:

The Methods section has been amended as shown below:

Ethics approval for pretesting was obtained (~~CUHREC~~ Curtin University Human Research Ethics Committee ~~0395/~~2019~~-0395~~) and participants provided written consent.

4. **RESULTS**

Feedback

- *In line 141-142, "While identical independent translations were rare, many of the differences in translation were not material." What's the meaning of "material"?*

Response:

We have amended the line as follows: “*While identical independent translations were rare, many of the differences in translation were not material in the sense that they did not change the intended meaning of the source text.”*

5. **DISCUSSION**

Feedback:

- *In the Discussion, it is stated that "Brislin's approach to back-translation was regarded as the gold standard". What's the justification for adopting other approach e.g. TRAPD for translation but not adopting Brislin's approach? What's the advantages of TRAPD over Brislin's approach?*

Response:

The Introduction has been revised as follows to address this earlier in the manuscript:

~~It is generally recognised that ‘team translation’ represents the best current method for translating surveys.[11]~~ The *Guidelines for Best Practice in Cross-Culture Surveys* recommend “a team translation approach for survey instrument production” noting that “[o]ther approaches, such as back translation, although recommended in the past, do not comply with the latest translation research” [11]. ‘Team translation’ is considered preferable to other approaches on the basis that it enables people with complementary knowledge and expertise (e.g. language and cultural experts, survey researchers, and people with expert knowledge relevant to the particular survey topic) to work together to arrive at the best translation to ensure that survey items convey what they were intended to convey.[12] In team translation, two independent translations are produced and are then compared (item-by-item) by bilingual reviewers who work with an adjudicator to identify the ‘best’ translation for pretesting.[11] However, there are relatively few published examples describing how team translation has been implemented in practice, and much of the available literature relates to translations carried out as part of large and relatively well-resourced surveys, such as the European Social Survey.[~~12-16~~13-17]

The Discussion has also been revised as follows:

Finally, it is worth remembering that our understanding of what constitutes best practice changes over time. ~~It was not long ago that~~ For decades, ~~Brislin’s approach to~~ back-translation was regarded as the gold standard.[~~32~~ 12] However, an over-reliance on back-translation is now considered prone to producing more literal translations which can overlook important conceptual or cultural nuances.[12, 34, 35] As Bretschneider and colleagues note, “[t]he term ‘best practice’ implies that it is best when compared to any alternative course of action”.[33] While the in-depth discussions that occur in team translation methods such as TRAPD are considered useful in detecting nuances in meaning and achieving equivalence between languages, ~~However~~ it has been noted empirical data on “the contribution of the TRAPD process … to the overall quality of the translated survey instrument”[24] remains limited, and studies comparing the results of different translation methods are even more rare. Consequently, it makes sense for researchers to remain open to exploring and gathering data on a variety of approaches to translation provided they are transparently reported and critically appraised.

6. **STYLE**

Feedback:

- *Please ensure that your manuscript meets PLOS ONE's style requirements, including those for file naming.*

Response:

All files have been re-uploaded to meet the requirements for file naming. All other style requirements have been reviewed and the following changes have been made:

- Line numbers added to title page;
- Title on title page centred and font size increased;
- Corresponding authors initials added in parentheses after email address;
- Abstract reformatted to appear as a single paragraph with first-line indentation;
- Figure 1 now cited as ‘Fig 1’;
- Full stops added to the end of Table titles.

7. **FINANCIAL DISCLOSURE**

Feedback:

- *We note that one or more of the authors is affiliated with the funding organization, indicating the funder may have had some role in the design, data collection, analysis or preparation of your manuscript for publication; in other words, the funder played an indirect role through the participation of the co-authors. If the funding organization did not play a role in the study design, data collection and analysis, decision to publish, or preparation of the manuscript and only provided financial support in the form of authors' salaries and/or research materials, please do the following:*

a) *Review your statements relating to the author contributions, and ensure you have specifically and accurately indicated the role(s) that these authors had in your study. These amendments should be made in the online form.*
b) *Confirm in your cover letter that you agree with the following statement, and we will change the online submission form on your behalf:
   “The funder provided support in the form of salaries for authors [insert relevant initials], but did not have any additional role in the study design, data collection and analysis, decision to publish, or preparation of the manuscript. The specific roles of these authors are articulated in the ‘author contributions’ section.*

Response:

We confirm the following statement:

The funder Curtin University provided support in the form of salaries for authors DV, MR, AR, and RL and scholarship support to NP, but did not have any additional role in the study design, data collection and analysis, decision to publish, or preparation of the manuscript. The specific roles of these authors are articulated in the ‘author contributions’ section.

8. **FIGURES**

Feedback:

- *Please remove your figures from within your manuscript file, leaving only the individual TIFF/EPS image files, uploaded separately.  These will be automatically included in the reviewers’ PDF.*

Response:

- The figure has been deleted in the revised manuscript.

We are grateful for the contributions you have made to help improve this paper. We hope that our revisions have adequately addressed the issues you have identified.

Yours sincerely,

The Authors
