# Supplementary material for: Translating best practice into real practice: Methods, results and lessons from a project to translate an English sexual health survey into four Asian languages
Source: PLoS One. 2021 Dec 17;16(12):e0261074. doi: 10.1371/journal.pone.0261074 (PMC8682877; doi:10.1371/journal.pone.0261074)
Supplement: Attachment — Submitted filename: Response to reviewers.docx [file pone.0261074.s005.docx]

November 2021

Dear Editors,

**Translating best practice into real practice: methods, results and lessons from a project to translate an English sexual health survey into four Asian languages**

Thank you for providing additional feedback on our manuscript. We have reproduced and responded to each item of feedback below. Red font indicates new material, and ‘strike through’ shows where material from the previous draft has been deleted.

**Introduction**

1. *The authors have attempted to address the comments, but the justification for some issues are not well supported, including … the strengths of adopting TRAPD model for instrument translation. [Editor]*

We amended the Introduction to further emphasise the strengths of team translation, one version of which is TRAPD:

There are several approaches to translation. Forward-only translation (also known as ‘direct’ or ‘one-for-one’ translation) involves a single individual translating an instrument from one language (the source language) into a second language (the target language).[11] While forward-only translation has the advantage of saving time and costs, it is considered problematic because it “involves a total dependence on the [single] translator’s skill and knowledge, and often results in low validity and reliability”.[12] Forward-backward translation (also known simply as ‘back translation’) represents an attempt to overcome the risks inherent in relying on a single individual. In forward-backward translation, a second individual translates the target language instrument back into the source language; the original source language instrument and the back-translated source language instrument are then compared, and any discrepancies serve as indications of the need for further refinements of the translation.[13] However, a criticism of forward-backward translation is that it has the potential to focus too narrowly on the task of literal translation at the expense of ensuring that the translation captures the intended meaning of the survey item in a way that is clear and suitable for the intended audience.[14] Similarly, Behr cites an example in which ‘care services’ was forward translated into German as *pflegedienste* and back translated as ‘care services’ suggesting no error, when in fact the translated term “did not fit the questionnaire context since it is only used in the context of the ill and/or the elderly and is thus not fitting to general child care services”.[15] Ozolins and colleagues have reported how some forward translators choose literal translations (despite their misgivings as to whether it actually captures the intended meaning) because they do not want their translation to be flagged as an ‘error’ by the back translator.[16]

Consequently, there has been a growing call for more nuanced and layered approaches to instrument translation in which accredited translators, other people who speak both the source and target languages, survey researchers, and subject matter experts work together to produce translated surveys which: (1) capture the intended meaning of the source instrument; (2) reflect the cultural and contextual specificities of the target population; and (3) will facilitate meaningful comparisons of data across populations.[13, 17-20] Indeed, the *Guidelines for Best Practice in Cross-Cultural Surveys* recommend “a team translation approach for survey instrument production” noting that “[o]ther approaches, such as back translation, although recommended in the past, do not comply with the latest translation research” [21]. ‘Team translation’ (also known as ‘committee translation’) is considered preferable to other approaches on the basis that it enables people with complementary knowledge and expertise ~~(e.g. language and culture, survey methodology, knowledge relevant to the particular survey topic)~~ to work together to arrive at the best translation to ensure that survey items convey what they were intended to convey to the target audience.[22]

While team translation can assume a variety of forms, the approach known as TRAPD (Translation, Review, Adjudication, Pretesting, and Documentation) is the version endorsed in the *Guidelines for Best Practice in Cross-Cultural Surveys.*[21] Under the TRAPD model, ~~In team translation~~ two independent translations are produced and are then compared (item-by-item) by bilingual reviewers who possess study design and subject-matter knowledge, ~~who~~ and work with an adjudicator to identify the ‘best’ translation for pretesting; each step is documented for transparency.[21, 23]

2. *I would suggest the authors to review the aim of this article – Is it a methodological discussion or research paper? If it focuses on the methodology, a more critical discussion is warranted to compare the TRAPD model with other translation approaches in terms of its reliability and practicality, with sharing of practical experience and insightful recommendations. If it is more research-focused, findings need to be presented in an analytical manner in respective to a well-defined research aim. [Reviewer]*

Thank you for this suggestion. We have revised the Introduction to more clearly communicate the intended aim and scope of our manuscript:

In this article, we apply the TRAPD model to translate an English-language sexual health and blood-borne virus survey into four languages for migrants living in Australia. The aim of the study is to:

(1) document how TRAPD can be applied in practice, including any challenges in its application;
(2) provide examples of issues identified through the processes of team-based ‘review and adjudication’ and pretesting, which are key features of the TRAPD model;
(3) offer guidance to future researchers who seek to use the TRAPD method; and
(4) provide recommendations for further research priorities on the subject of survey translation.

The aim is not to empirically test the effectiveness of TRAPD relative to other methods of translation.

The study makes an important contribution to the literature since there are ~~However, there are~~ relatively few published examples describing how TRAPD ~~team translation~~ has been implemented in ~~practice~~ the context of survey research, despite it being the model endorsed in the *Guidelines for Best Practice in Cross-Cultural Surveys*.~~, and m~~ Much of the available TRAPD literature relates to translations carried out as part of large and relatively well-resourced surveys, such as the European Social Survey.[24-28] However, as Sha and Lai have argued ~~that~~ “[i]t is important to identify a viable translation process that can be adapted and tailored to the varying level of expertise and resources available”.[29] ~~The purpose of this paper is to document the process, results and lessons from a project to translate an English-language survey into four languages for migrants living in Australia. The project formed one part of study to ascertain the feasibility of conducting a periodic national survey in Australia to measure migrants’ knowledge, attitudes and practices in relation to sexual health and blood-borne viruses.~~

Amendments have also been made to the Results and Discussion sections of the paper to more explicitly reflect the aims described above.

**Methods**

3. *“The authors have further explained the reason for recruiting a relatively small sample for pretesting, which is understandable. While budget is limited, I notice that the number of respondents is disproportionate among different communities. Only 3 Chinese (3.8% of the estimated population size) but 20 Vietnamese (66.7%) were recruited. This difference cannot be solely explained by budgetary constraint. Were there any practical reasons?” [Reviewer; editor also requested justification for the disproportionate sample size]*

We have provided the following additional explanation:

The larger sample size for Vietnamese pretesting was opportunistic in the sense that a group of 20 participants were gathered for another purpose and expressed willingness to provide feedback on the translated instrument; although this resulted in the Vietnamese pretest sample being larger than those representing other language groups, the opportunity to obtain more feedback with minimal additional resources was recognised as an efficient means of obtaining more data to check instrument validity. There was no intention to engage in any statistical comparison of differences in pretest responses between the communities.

4. *Please explain why simplified Chinese does not need to be reviewed separately? In fact, you mentioned that differences existed in Chinese used in Mainland China, Taiwan, and Hong Kong (in fact, there are other major communities using Chinese).*

As stated in the Introduction, the four languages for translation using the TRAPD method were Traditional Chinese, Karen, Khmer and Vietnamese. Translation into Simplified Chinese using the TRAPD method was not a stated aim of this research and, consequently, the results from the Simplified Chinese adaptation process are not presented in the Results section (or in Appendix S6). We have decided to remove the reference to Simplified Chinese in the Methods section to avoid confusion. The process of adapting the Traditional Chinese survey into Simplified Chinese was distinct from the TRAPD method and, as such, does not fall within the scope of the current paper; it is better suited to a separate manuscript on the subject of the process of adapting translated survey tools for use with different linguistic communities/dialects.

**Findings**

5. *In response to Question 4 (“Have the authors made all data underlying the findings in their manuscript fully available?”) the Reviewer has answered “No”.* *[Reviewer]*

We note that we have provided raw data in the document marked Appendix 3 – TRAPD results. The results for each language are included in a separate workbook within the Excel file (screen shot below).

6. *Were findings of all languages presented in the Results section? I notice that much of the results focuses on the Traditional Chinese version. [Reviewer]*

We note that the results for each language are included in a separate workbook within the Excel file marked Appendix 3 – TRAPD results (see above). We have also significantly amended the structure of the Results section, including the introduction of a new Table 5 (below) to highlight examples from all four languages.

| **Items / key terms** | **Language** | **Translation 1** | **Translation 2** | **Preferred translation** |
| --- | --- | --- | --- | --- |
| Chlamydia | Vietnamese | Bệnh hoa liễu | Bệnh lậu (khuẩn Chlamydia) | Bệnh Chlamydia |
|  |  | *English meaning: Venereal disease* | *English meaning: Gonorrhoea (chlamydia bacteria)* |  |
|  | Traditional Chinese | 衣原體 | 衣原體病 | 衣原體 (披衣菌) 感染 |
|  |  | *English meaning: Chlamydia* | *English meaning: Chlamydia infection/sickness* | Chlamydia in two dialects + term for infection |
| Gonorrhoea | Vietnamese | Bệnh lậu | Bệnh da liễu | Translation 1 |
|  |  | *English meaning: Gonorrhoea* | *English meaning: Venereal disease* |  |
| HIV | Traditional Chinese | 愛滋病病毒 | HIV | Combine -  愛滋病病毒 (HIV) |
|  |  | *English meaning:* AIDS virus | *English meaning:* HIV |  |
|  | Karen | တၢ်ဆါဃၢ်ဒုးကဲထီၣ်အ့း(စ)တၢ်ဆါ (HIV) | HIV | Translation 1 |
|  |  | *English meaning:* Human Immunodeficiency Virus (HIV) | *English meaning:* HIV |  |
| Questions about sexual activities | Karen | တၢ်သံကွၢ်ဘၣ်ဃးသွံၣ်ထံးအတၢ်ဟူးတၢ်ဂဲၤတဖၣ်ဒီးတၢ်ရ့လိာ်မုာ်လိာ် | တၢ်သံကွၢ်တဖၣ်ဘၣ်ဃးမုၣ်ခွါသွံၣ်ထံးအတၢ်ဟူးတၢ်ဂဲၤဒီးတၢ်ရ့လိာ်သးသ့ၣ်တဖၣ် | Translation 1 |
|  |  | *English meaning: Questions about sex* | *English meaning: Questions about sex among men and women* |  |
| A casual sex partner | Vietnamese | Một người bạn tình không thường xuyên | Bạn tình bình thường (không có mối quan hệ ràng buộc) | Translation 2 |
|  |  | *English meaning: An occasional lover* | *English meaning: Casual partner (no tie relationship)* |  |
|  | Traditional Chinese | 臨時性伴侶 | 隨意的性伴侶 | 非固定性伴（没有稳定關係或臨時性伴，包括一夜情人 |
|  |  | *English meaning: Temporary partner* | *English meaning: Casual sex partner* | *Non-regular sexual partners (no stable relationships or temporary sexual partners, including one-night lovers)* |
|  | Khmer | ដៃគូរួមភេទដែលមិនមានទំនាក់ទំនងប្តូរផ្តាច់ជាមួយ | ដៃគូររួមភេទម្តងម្តាល | Translation 2 |
|  |  | *English meaning: Unprotected sexual partner* | *English meaning: Occasional sexual partners* |  |
| Someone you are in a committed relationship with (e.g. husband / wife, boyfriend / girlfriend) | Karen | ပှၤတဂၤလၢနအၢၣ်လီၤလၢနကအိၣ်ဃုာ်အီၤလၢသးတံၢ်နီၢ်(အဒိ-၀ၤ,မါ,တၢ်အဲၣ်တီခွါ,တၢ်အဲၣ်တီမုၣ်) | ySRw*R*RvXetd.’D;w>b.xGJ vXttd.’D;w>tX.vDRtDvDR (t’d = rgA0R< w>tJ.wDrk.Aw>tJ.wDcGg) | Translation 2 |
|  |  | *English meaning:* ‘Committed relationship’ translated as *‘willing – in mind- to live together’* | *English meaning:* ‘Committed relationship’ |  |
|  | Khmer | នរណាម្នាក់ដែលអ្នកមានទំនាក់ទំនងប្តូរផ្តាច់ជាមួយ (ឧទាហរណ៍ ប្តី / ប្រពន្ធ មិត្តប្រុស / មិត្តស្រី) | មនុស្សម្នាក់ដែលអ្នកមានទំនាក់ទំនងជាមួយច្បាស់លាស់ (ឧទាហរណ៍ ប្តី/ប្រពន្ធ, មិត្តប្រុស/មិត្តស្រី) | Translation 2 |
|  |  | *Someone you have a breakup with (for example, spouse / boyfriend / girlfriend)* | *Someone you have a clear relationship with (e.g. spouse, boyfriend / girlfriend)* |  |
| Did you use a condom the MOST RECENT time you had sex? (Tick one) | Karen | ဘၣ်မနုၤအဃိန တသူ ၀ဲဒၣ်တၢ်ဖျီၣ်(condom)တခါဖဲနအိၣ်ဃုာ်လိာ်နသးလၢခံကတၢၢ်တဘျီအခါန့ၣ်လဲၣ်. | zJerHCkm tvD>cHuwX>wbsD M.< b.rEkRCdvX ewpl;ug xh.zsD.vJ.I *(wdReD.Cmw>vX tb.xGJcJvXm)* | Translation 2 |
|  |  | *English meaning:* Condom is translated as *cover of this* | *English meaning:* Condom translated as cover of penis |  |
| How old are you? | Karen | အသးအိၣ်ထဲလဲၣ်. *(တိၤနီၣ်တခါ)* | eo;eH.td.ySJReH.vJ.I *(wdReD.Cmwcg)* | Translation 2 |
|  |  | Dialect of Irrawaddy Region | Dialect of Karen State |  |

**Discussion**

7. *“I think the numbering of paragraphs is not necessary, especially when some points are actually related (e.g., #6 and #7).” [Reviewer]*

Thank you for this suggestion. We have now removed the numbering from the paragraphs. The Discussion section has been significantly redrafted in light of the feedback (point 2 above) that *“a more critical discussion is warranted to compare the TRAPD model with other translation approaches in terms of its reliability and practicality, with sharing of practical experience and insightful recommendations.”*

8. *“[A]fter revisiting the discussion, I have questions about the advantages of TRAPD model – (i) In this study, the ‘leaner’ TRAPD model could save time and resources for translation. However, is there evidence that the outcome of TRAPD model (e.g., culture adaptability and language) is comparable to the original TRAPD model and the conventional approach?” [Reviewer]*

We have added the following paragraphs in our significantly reworked Discussion:

Whereas much of the existing TRAPD literature reports on the use of the method in the context of large and relatively well-resourced, international surveys, this study documents the experience of applying the TRAPD method to a modestly-resourced survey in one country. The purpose of this study is not to provide evidence that TRAPD is the final word in effective translation methods. As has been noted elsewhere, empirical data on “the contribution of the TRAPD process … to the overall quality of the translated survey instrument”[18] remains limited, and more studies comparing the results of different translation methods are needed. Instead, this paper shows that the TRAPD method has value in identifying errors and exposing nuances in translation, but that it can be difficult to implement in practice, particularly where resources are low and translations into many languages are required. As Knight and colleagues acknowledge, the reality of studying minority populations is that “research context often makes full compliance with … best practice recommendations impossible and … the best one can do is to make incremental approximations of these recommendations.”[34]

As was reported in the Methods, deviations from TRAPD ‘best practice’ occurred. Significantly, it was not possible to employ or compensate translation reviewers; instead, the task of reviewing was undertaken in-kind which made it difficult both to recruit multiple reviewers for each language, and to rigidly proscribe the manner in which the translation reviews were undertaken. For instance, while reviewers were instructed to code translation errors using the European Social Survey (ESS) Verifier Intervention Categories, few reviewers used the ESS categories, and some did not document any reasons for preferring one translation over another which made the process of adjudication difficult. Problems around documentation in the TRAPD review process have been noted in other studies, including the European Social Survey where it was noted that “maintaining documentation can be burdensome … The documentation provided by the country teams on the development of ESS translations was at times meagre.”[21]

In addition to being time-consuming, the practice of coding translation errors may not be familiar to bilingual reviewers who do not necessarily possess a research background. Many of the distinctions between the categories – for instance, register/wording issues, grammar/syntax issues and minor linguistic defects (which can include grammar) – are subtle or require a high level of linguistic awareness. This suggests that the use of alternative coding protocols should be considered and tested, and efforts should be made to simplify the process of documenting reasons for decision at each stage of the translation process. In an effort to understand the difficulties they experienced in obtaining adequate documentation from translation partners, Harkness, Villar and Edwards surmised “[t]his may be because those involved were not familiar with how and what to document, but it is also likely that the effort involved in manual documentation played a role.”[21] One solution may be for a member of the research team to assume responsibility for documentation by sitting with each panel member (either physically or virtually) while they independently review the translations. The researcher could record the preferred translation for each item and the reviewer’s reasons, using probing questions where necessary; this was the approach taken for the Karen translation in our study. An alternative would be to investigate the development and use of specialised software to manage the documentation process. It is noted that an online, interactive Translation Management Tool was developed for the Survey of Health, Ageing and Retirement in Europe (SHARE) and tested in round 8 of the European Social Survey in 2016, although a number of areas for improvement were identified.[21] Currently, the tool is not publicly available but can be tailored to support specific survey requirements at a cost.

It is not possible to determine whether closer adherence to TRAPD ‘best practice’ in this study would have improved the quality of the resulting translations. A literature review of studies using multi-step, team-based translations of health quality of life questionnaires found that “[a]lthough there is some evidence that different methods (i.e., ‘light/simpler’ vs. ‘heavy/complex’) yield similar results, this has been tested empirically only on a very limited scale” [12]. However, our study does demonstrate that even ‘light/simpler’ adaptations of the TRAPD method can successfully identify issues that may not have been apparent had non-team-based or single-round translation approaches been adopted. For instance:

- relying on only one translator to conduct a forwards-only translation without further review would have resulted in important errors being overlooked (e.g. the Khmer translation of ‘casual sexual partner’ as ‘unprotected sexual partner’, and the Karen translation of ‘committed relationship’ as ‘willing in mind to live together’);
- relying on forward-backward translation only may not have revealed importance nuances (e.g. the translation of hepatitis B as 乙型肝炎 would likely have been translated back into English as ‘hepatitis B’ suggesting no problem with the translation, when in fact the review process highlighted that it would not be inclusive of/familiar to Taiwanese-Chinese speakers);
- not including researchers as partners in the translation process may have resulted in translations which did not reflect the intended meaning of the survey items (e.g. a bilingual reviewer without a sexual health and blood-borne virus background favoured the narrow, heteronormative translation of Karen translation of ‘sexual activities’ which meant ‘sex among men and women’);
- pretesting assisted in identifying typographical/formatting errors made when incorporating the reviewed translations into the survey tools (e.g. the omission for the term ‘no/not’ in one item in the Karen survey, and parts of the Karen script not being displayed correctly);
- the benefits of assembling a team comprising people with complementary expertise was particularly evident when attempting to translate technical, medical terminology in a way that, on the one hand, is meaningful to the target audience and, on the other hand, guards against perpetuating terms which are stigmatising or give rise to inaccurate assumptions (e.g. the difference between ‘clinical’ and ‘lay’ translations of HIV into Traditional Chinese).

It is, of course, possible that some errors in translation were not detected as a result of the approach we adopted. For this reason, we have taken the rare step of making all of our translation data publicly available (Appendix S3)…

9. *“Regarding the rigour, I notice that the TRAPD model was not adhered thoroughly. For example, some reviewers did not provide their comments for adjudication and documentation was simplified. Would the failure to adhere suggest the difficulty maintaining the rigour of the model in practice? (As you argued that the TRAPD model is the ‘real practice’)” [Reviewer]*

The Reviewer is correct to identify our conclusion that it may not be possible to rigidly apply TRAPD in all contexts. As noted in our response to Comment 8 (above), we argue that ‘light/simpler’ adaptations may be necessary and justifiable, and that more research needs to be undertaken on this point.

10. *“The authors suggested additional research on the application of TRAPD model in translating survey instruments. Why kind of research or data do they recommend?” [Reviewer]*
11. *“discuss the impact and significance of this study on future research.” [Editor]*

We have responded to Comments 10 and 11 through the addition of the following paragraphs:

Given the dearth of clear empirical evidence about the most accurate and feasible method of undertaking translations, we encourage future researchers to follow our example. Greater transparency around survey translation research processes and results will: (1) allow the accuracy of different approaches to be studied in greater detail; (2) provide practical insight into effective ways of adapting established processes in response to different needs, settings and levels of resourcing; and (3) enable the validity of the survey results to be assessed with reference to the quality of the underlying translations.

Unless a culture of transparent trial-and-error in translation is fostered, negative consequences will follow. First, survey researchers may feel daunted by the complexity (and uncertainty) of translation and may therefore avoid attempts to engage with linguistically diverse populations; this would either result in: (a) underrepresented sections of the population continuing to be overlooked in studies geared towards collecting data to improve health outcomes, or (b) respondents having no option but to complete surveys in a language other than their preferred language, thus increasing the potential for survey items to be misunderstood and data quality to be compromised. Second, survey researchers who have attempted to undertake translations but whose methods have deviated from ‘best practice’ (e.g. due to circumstantial challenges like budget limitations) may hesitate to publish their translation processes and results for fear of being criticised; the consequence of this would be a continued dearth of information about the most effective and efficient means of undertaking survey translations. As a recent editorial in Nature Human Behaviour makes plain, “[s]cience is messy, and the results of research rarely conform fully to plan or expectation. ‘Clean’ narratives are an artefact of inappropriate pressures and the culture they have generated.”[36] It is only through more detailed accounts of the processes and results of survey translation that we can come closer to understanding which methods (and variations of methods) are best suited to produce the most accurate results in specific contexts. Given the increase in the scale of international (pre-pandemic) migration,[37] and the growing appetite for multinational, multicultural and multiregional surveys,[38] the importance of continuing to build the evidence-base for survey translation is both apparent and urgent.

We trust that these amendments adequately address your comments and we are grateful for the role you have played in strengthening our manuscript.

Yours sincerely,

The authors
