# Supplementary material for: Translating best practice into real practice: Methods, results and lessons from a project to translate an English sexual health survey into four Asian languages
Source: PLoS One. 2021 Dec 17;16(12):e0261074. doi: 10.1371/journal.pone.0261074 (PMC8682877; doi:10.1371/journal.pone.0261074)
Supplement: Attachment — Submitted filename: Response to reviewers.docx [file pone.0261074.s006.docx]

22 November 2021

Dear Editors,

**Translating best practice into real practice: methods, results and lessons from a project to translate an English sexual health survey into four Asian languages**

Thank you for providing additional feedback on our manuscript. We have reproduced and responded to each item of feedback below.

**FEEDBACK FROM EDITOR**

1. *In the abstract, in Line 44 and 45, the sentence "...TRAPD method can identify issues that may not have been apparent had non-team-based or single-round translation approaches have been adopted" is difficult to understand. Please rephrase and revise.*

Both the unmarked and tracked versions of the manuscript had the correct wording, as follows:

However, our study does demonstrate that even adaptations of the TRAPD method can identify issues that may not have been apparent had non-team-based or single-round translation approaches been adopted.

2. *In Line 116, the sentence "the aim is not to empirically test the effectiveness of TRAPD relative to other methods of translation." can be deleted.*

We have deleted the sentence.

3. *I would like to clarify that supplementary files including S1, S2 and S3 will not be included in the published manuscript.*

We understand from PLOS ONE guidelines that:

Supporting information is auxiliary to the main content of the article. In the online version of the published article, readers access the files via hyperlinks in the Supporting Information section of the article. PLOS hosts these files on its servers and also deposits them on Figshare to increase compliance with the FAIR principles of data sharing. Supporting information files are published exactly as provided, and are not copyedited … The PLOS publishing platform supports any file type for supporting information files.

We are happy for the supplementary files to be accessible via hyperlinks to the documents hosted on the PLOS servers/Figshare. However, if you require us to provide the files in a different format, or to host them on our project website, please let us know so that we can accommodate your request.

4. *In the section or pre-testing, in Line 205-219, "The minimum target sample size ...... who would likely to speak English not well or not at all is unknown" should be deleted as the calculated sample size was not achieved and not related to the pre-testing survey.*

We have deleted these lines as indicated, and amended reference numbers accordingly:

Ethics approval for pretesting was obtained (Curtin University Human Research Ethics Committee 2019-0395). The minimum target sample size for the pilot survey (in any language) was 1,116 respondents equally divided between the three regions of birth (Sub-Saharan Africa, North East Asia and South-East Asia) to detect regional differences at a significance level of 5% and 90% power. ~~Based on available Census data on country of birth and English-language proficiency, it was estimated that:~~

- ~~82.00% (n=305) of North East Asian-born respondents would be from a country in which Chinese was the dominant language and that, assuming all spoke some Chinese, 25.9% (n=79) would be likely to not speak English well or at all;~~
- ~~25.21% (n=94) of South East Asian-born respondents would be from Vietnam and that, assuming all spoke Vietnamese, 31.50% (n=30) would be likely to not speak English well or at all;~~
- ~~3.81% (n=14) of South East Asian-born respondents would be from Cambodia and that, assuming all spoke Khmer, 33.50% (n=5) would be likely to not speak English well or at all;~~
- ~~3.75% (n=14) of South-East Asian-born respondents would be from Myanmar and less than 10% would speak Karen (proportion of those who would be likely to speak English not well or not at all is unknown) [32,33].~~

Given ~~these small numbers,~~ the limited availability of resources for pretesting, the exploratory nature of the feasibility study, and the fact that multiple quality checking methods were built into the study design (e.g. two independent translations, review and adjudication), a pragmatic decision was made to only pretest translations on small samples

5. *In Table 4, it is shown that 9 questions resulted in identical independent translations, but in Line 249, it is stated that 12 were identical. Please clarify and revise accordingly.*

Thank you for pointing this out; it should read ‘nine’; we have amended accordingly.

**FEEDBACK FROM REVIEWER 1**

6. *One suggestion is to add a brief conclusion at the end of the manuscript.*

We have added the following sub-section:

**Conclusions**

Survey translation is time-consuming and costly and, as a result, deviations from TRAPD ‘best practice’ occurred. It is not possible to determine whether closer adherence to TRAPD ‘best practice’ would have improved the quality of the resulting translations. However, our study does demonstrate that even adaptations of the TRAPD method can identify issues that may not have been apparent had non-team-based or single-round translation approaches been adopted. Given the dearth of clear empirical evidence about the most accurate and feasible method of undertaking translations, we encourage future researchers to follow our example of making translation data publicly available to enhance transparency and enable critical appraisal.

We trust that these amendments adequately address your comments. Please advise whether you require anything further from us.

Yours sincerely,

The authors
